# Hyperpolarized ^13^C NMR Reveals Pathway Regulation in *Lactococcus lactis* and Metabolic Similarities and Differences Across the Tree of Life

**DOI:** 10.3390/molecules29174133

**Published:** 2024-08-30

**Authors:** Sebastian Meier, Alexandra L. N. Zahid, Lucas Rebien Jørgensen, Ke-Chuan Wang, Peter Ruhdal Jensen, Pernille Rose Jensen

**Affiliations:** 1 Department of Chemistry, Technical University of Denmark, 2800 Kongens Lyngby, Denmark; 2 Department of Health Technology, Technical University of Denmark, 2800 Kongens Lyngby, Denmark; alza@dtu.dk (A.L.N.Z.); jorgensenlucas96@gmail.com (L.R.J.); kcwan@dtu.dk (K.-C.W.); 3 Department of National Food Institute, Technical University of Denmark, 2800 Kongens Lyngby, Denmark; perj@food.dtu.dk

**Keywords:** ^13^C NMR, glycolysis, dDNP NMR, in-cell NMR, kinetic control, *Lactococcus lactis*, substrate mixtures

## Abstract

The control of metabolic networks is incompletely understood, even for glycolysis in highly studied model organisms. Direct real-time observations of metabolic pathways can be achieved in cellular systems with ^13^C NMR using dissolution Dynamic Nuclear Polarization (dDNP NMR). The method relies on a short-lived boost of NMR sensitivity using a redistribution of nuclear spin states to increase the alignment of the magnetic moments by more than four orders of magnitude. This temporary boost in sensitivity allows detection of metabolism with sub-second time resolution. Here, we hypothesized that dDNP NMR would be able to investigate molecular phenotypes that are not easily accessible with more conventional methods. The use of dDNP NMR allows real-time insight into carbohydrate metabolism in a Gram-positive bacterium (*Lactoccocus lactis*), and comparison to other bacterial, yeast and mammalian cells shows differences in the kinetic barriers of glycolysis across the kingdoms of life. Nevertheless, the accumulation of non-toxic precursors for biomass at kinetic barriers is found to be shared across the kingdoms of life. We further find that the visualization of glycolysis using dDNP NMR reveals kinetic characteristics in transgenic strains that are not evident when monitoring the overall glycolytic rate only. Finally, dDNP NMR reveals that resting *Lactococcus lactis* cells use the influx of carbohydrate substrate to produce acetoin rather than lactate during the start of glycolysis. This metabolic regime can be emulated using suitably designed substrate mixtures to enhance the formation of the C4 product acetoin more than 400-fold. Overall, we find that dDNP NMR provides analytical capabilities that may help to clarify the intertwined mechanistic determinants of metabolism and the optimal usage of biotechnologically important bacteria.

## 1. Introduction

While the canonical Embden–Meyerhof–Parnas (EMP) glycolysis pathway and its steps have long been identified, mechanistic details including its intracellular thermodynamics, kinetics and responsiveness have remained incompletely understood. These shortcomings have recently been challenged by suitably developed methodology including concentration measurements, flux analysis, and thermodynamic analysis [1,2], alongside non-invasive methods to visualize kinetic barriers during substrate influx into central reaction networks [3,4]. Restraints to pathway chemistry include the physicochemical properties of pathway intermediates, such as low toxicity and their utility as precursors, in addition to thermodynamic favorability [5]. Determination of metabolic energetics across the tree of life has indicated that absolute metabolite concentrations and Gibbs free energies (Δ*G*) are largely conserved [1]. Irreversible steps of EMP glycolysis with large negative Δ*G*, especially at the phosphofructokinase and pyruvate kinase-catalyzed steps, have been postulated as a rule of thumb to provide reactions with plausible control of glycolysis [6]. The direct experimental probing of the accumulation of intermediates in thermodynamically favored but kinetically slow metabolic pathway reactions poses challenges, as it requires the use of rapid noninvasive methodology. 

Hyperpolarized NMR spectroscopy has emerged as a uniquely suitable method to rapidly probe reactions inside living cells [7,8,9,10,11,12,13] or other complex environments [14,15]. The method uses the temporary redistribution of nuclear spin states to drastically, albeit temporarily, enhance nuclear spin polarization. This enhancement can exceed four orders of magnitude for suitable preparations of hyperpolarized substrates. The method is currently limited to fast processes, as the enhanced spin polarization fades by the relaxation time T_1_, which reaches 10–50 s for non-protonated carbons in common metabolites. The resultant time window is sufficient to probe the uptake and conversion of rapidly transported and metabolized substrates in the natural, intracellular environment [16]. The use of hyperpolarized hexoses with ^13^C-labeled non-protonated sites provides substrates with T_1_ times of the order of 10–30 s [17,18] and can be achieved using the microwave-driven transfer of spin alignment from electrons in stable radicals to the nuclei of metabolic substrates at low temperatures (~1 K) in a process called dynamic nuclear polarization (DNP), which is conducted in a dedicated polarizer [7]. The substrate can be washed out of the polarizer using hot buffer to result in hyperpolarized substrate that has been hyperpolarized by a process that is known as dissolution DNP (dDNP) (Figure S1). Hyperpolarized hexoses address the sensitivity limitations of conventional NMR reaction tracking [19] and have been used to track the dynamics of central metabolism, specifically in glycolysis, the pentose phosphate pathway (PPP) and initial steps in the Krebs cycle in *E. coli* [20], various yeasts of biotechnological importance [21], and human cells [9,10,17,22]. These measurements have often focused on the different evident outputs of glycolysis. Due to signal overlaps with the strongly enhanced substrates, the metabolites of upper glycolysis, which resemble the substrate more closely than products of catabolism, have not systematically been identified and compared in different cells.

Here, we set out to visualize the central metabolism of *Lactococcus lactis* (*L. lactis*, MG1363), a generally regarded as safe (GRAS) Gram-positive bacterium of central biotechnological importance. *L. lactis* has probiotic properties and is widely used in the production of fermented dairy products, recombinant proteins and metabolites [23]. Previous characterization of *L. lactis* metabolism has widely employed conventional NMR spectroscopy for steady-state reaction tracking [24,25] and metabolomic analyses [26], while direct insight into glycolytic kinetics using a pulse of hyperpolarized carbohydrates has not been reported. In fact, no hyperpolarized in-cell NMR metabolic studies of glycolytic carbohydrate metabolism have been reported for Gram-positive bacteria to the best of our knowledge, thus raising the question of whether their envelope or intracellular ion concentrations obstruct such pathway observations. By contrast, the metabolism of hyperpolarized pyruvate in Gram-positive *Staphylococcus aureus* has been previously studied [27]. 

Here, we hypothesized that hyperpolarized ^13^C NMR can complement other (conventional) NMR measurements and provide insights into kinetic barriers in the carbohydrate metabolism of Gram-positive bacteria, its regulation and its malleability upon genomic and nutritional intervention. We find that direct observations of extended pathways in the glycolytic conversion of hyperpolarized carbohydrates in *L. lactis* are viable. The main kinetic intermediates in glycolysis include the fructose-6-phosphate and fructose-1,6-bisphosphate species in addition to precursors of biomass such as dihydroxyacetone phosphate, 3-phosphoglycerate and pyruvate. Overflow at the pyruvate nexus following a glucose pulse leads to a significant formation of acetoin, a fragrance and flavor compound, on a short timescale of seconds. Site-specifically isotope enriched, hyperpolarized substrate was used to compare the kinetic barriers in upper glycolysis of *L. lactis* to those of *E. coli*, *S. cerevisiae*, and human cancer cells. The rate of conversion of glucose through upper glycolysis increased in pre-steady-state kinetics in the order *L. lactis* < *E. coli* < *S. cerevisiae* < human cancer cells. The ratio of fructose-6-phosphate to fructose-1,6-bisphosphate that formed rapidly from a glucose pulse, and hence the kinetic barrier created by the phosphofructokinase reaction, correlated with the overall glycolytic progress in the different cell types. The overflow of pyruvate into acetoin could be exploited in suitable glucose/pyruvate substrate mixtures [4], which can modify the selectivity of *L. lactis* metabolism drastically to yield acetoin on a par with lactic acid formation in the absence of genetic engineering. 

## 2. Results and Discussion

### 2.1. DNP NMR Using Hyperpolarized [U-^13^C,^2^H]glucose in L. lactis

**Scheme 1:**
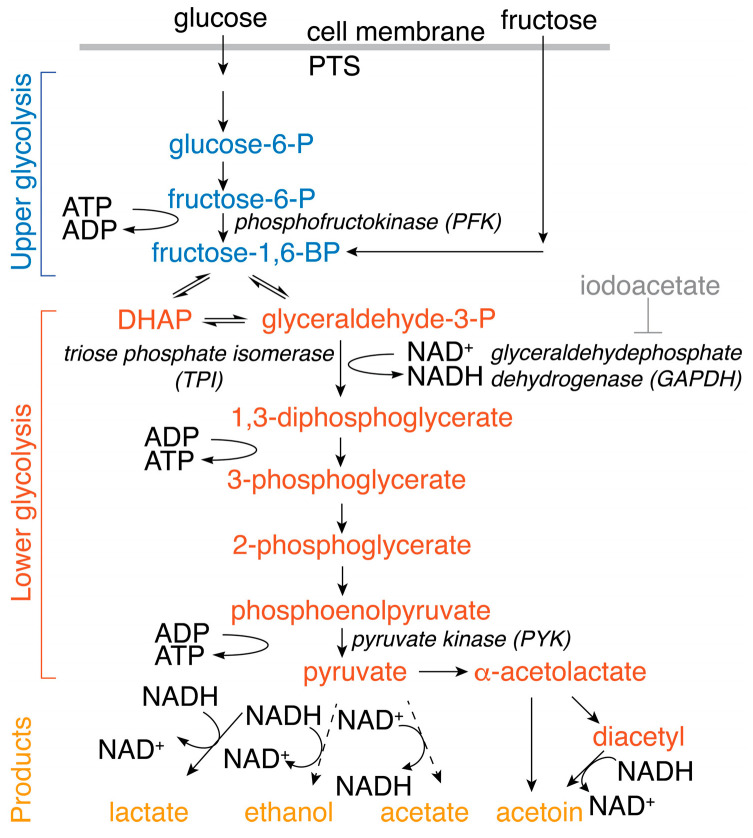
Schematic overview of Embden–Meyerhof–Parnas glycolysis commencing from glucose (taken up via the phosphotransferase system (PTS)) or fructose, and of pyruvate metabolism to fermentation products in *L. lactis.* The effects of inhibition by iodoacetate were probed alongside the effects of changing TPI, GAPDH and PYK enzyme levels.

*L. lactis* MG1363 is a wild-type strain that is among the most used lactic acid bacteria for physiological studies. *L. lactis* is a homofermentative bacterium of industrial importance with a “generally regarded as safe” status [28,29]. Despite its relevance in bio-technology, the modeling of the convoluted intricacies and interdependencies in *L. lactis* metabolism has been considered work in progress [24,30]. The validation of models is limited as computational mechanistic models remain hard to probe for their predictive value in vivo in the absence of counterpart experimental data. Experimental input from conventional in-cell NMR metabolic studies has contributed significantly to the understanding of *L. lactis* metabolism [24,25,30,31,32,33,34], albeit insight into kinetic barriers and rapid processes remains sparse. Previous studies have indicated a kinetic barrier at the pyruvate kinase step, and the accumulation of upstream fructose-1,6-bisphosphate due to ATP excess and limitation of oxidized nicotinamide adenine dinucleotide (NAD^+^; Scheme 1) [35]. Here, we explored the dynamic response of *L. lactis* using dDNP ^13^C NMR. We set out to follow the fate of hyperpolarized [U-^13^C,^2^H]glucose that was rapidly injected into a suspension of *L. lactis* cells. Such assays had previously provided mechanistic details regarding the start of glycolysis upon a bolus of hyperpolarized [U-^13^C,^2^H]glucose in yeasts, *E.coli* and human cell cultures [9,10,17,20,22], but there have been no applications to Gram-positive cells.

**Figure 1:**
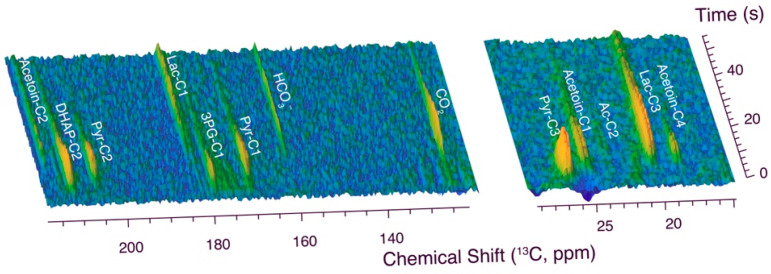
Real-time observation of glucose influx into metabolites of *L. lactis* (MG1363) at 303 K. Hyperpolarized [U-^13^C,^2^H]glucose (6 mM) was injected into a suspension of *L. lactis* and conversion of the glucose probe molecules was tracked using a time series of ^13^C NMR spectra implemented as a pseudo-2D data set, acquiring one spectrum per 500 milliseconds using a 10° excitation pulse. Detected carbon sites are indicated as C1–C4. Abbreviations: 3PG—3-phosphoglycerate; Ac—Acetate; DHAP—dihydroxyacetone phosphate; Lac—lactate; Pyr—pyruvate.

The conversion of hyperpolarized [U-^13^C,^2^H]glucose by wild-type *L. lactis* MG1363 on a timescale of seconds is displayed in Figure 1 (Figure S1 shows a detailed description of the experimental setup). Assignments in hyperpolarized ^13^C NMR spectra need to rely primarily on chemical shifts and were based on an accurate and pH-dependent collection of reference compound assignments [4,22]. The conversion of glucose by wild-type *L. lactis* MG1363 was fast enough to detect metabolites accumulating on a timescale of a few seconds and to thus enable the detection of glucose influx into the glycolysis of resting *L. lactis*. Major metabolites accumulating due to kinetic barriers include dihydroxyacetone phosphate (DHAP), 3-phosphoglycerate (3PG), and pyruvate (Pyr). The conversion of these intermediates has been previously identified as a major bottleneck in the metabolisms of prokaryotic as well as eukaryotic model organisms. A common feature of the resultant accumulating metabolites is their role as non-toxic precursors for biomass, including lipids and several amino acids [5]. Hence, the formation of materials seems to constitute a constraint on the kinetic control principles of the metabolic process. In addition to the accumulation of the aforementioned intermediates, the formation of acetoin and CO_2_/HCO_3_^–^ was observed on the timescale of seconds, followed by the slower formation of lactic acid as the main product, which dominated after metabolic adaptation occurring within one minute of a glucose pulse. Hence, dDNP NMR provides insight into mechanistic changes during the start of *L. lactis* glycolysis upon a bolus of hyperpolarized [U-^13^C,^2^H]glucose on a timescale that is complementary to the timescale of conventional NMR spectroscopy (minutes to hours). These observations show that the rapid availability of glucose in *L. lactis* can lead to an overflow of carbon into precursors for biomass and into uncharged C4 compounds prior to the formation of lactic acid. The conversion of glucose through entire glycolysis in *L. lactis* was sufficiently rapid on the T_1_ timescale to allow detection of end products with dDNP NMR. However, the accumulation of these products after approximately 20 s was significantly slower than corresponding glycolytic conversions using *E. coli*, *S. cerevisiae* and other yeasts or human cancer cell lines. Hence, glycolytic kinetics appeared to be less optimized in *L. lactis*, a notion that we chose to investigate using hyperpolarized site-specifically labeled substrates.

### 2.2. Hyperpolarized [1-^13^C,^2^H]glucose Probes Upper Glycolysis in L. lactis and Other Model Cell Lines

**Figure 2:**
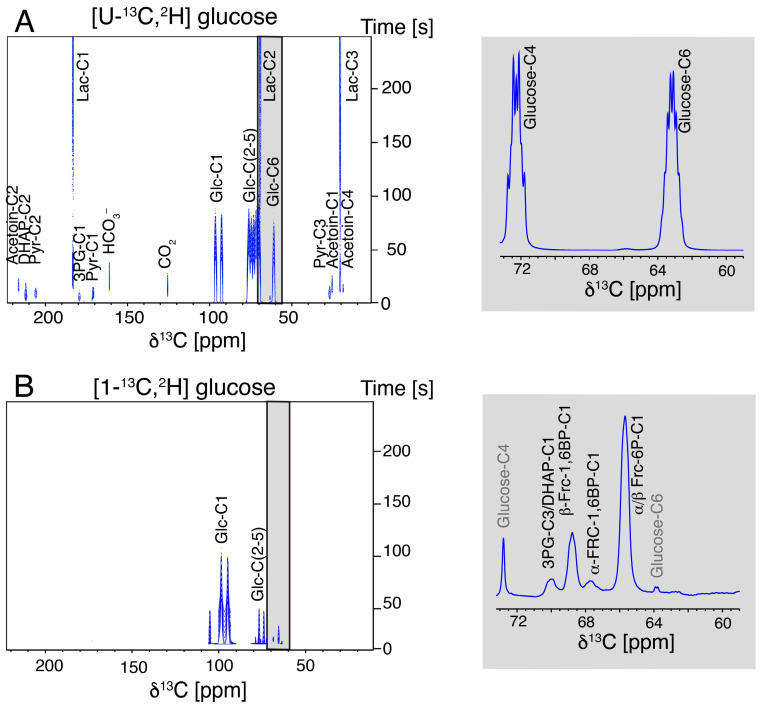
Comparison of the real-time observation of glucose influx into metabolites of *L. lactis* (MG1363) at 303 K using (**A**) hyperpolarized [U-^13^C,^2^H]glucose and (**B**) hyperpolarized [1-^13^C,^2^H]glucose. Grey inserts display the sums of the first 40 spectra in the time course.

Due to the chemical similarity of the hyperpolarized glucose substrate and the phosphorylated glucose and fructose metabolites of upper glycolysis, these metabolites are hard to detect against the background of the substrate (Figure 2A). Hence, we employed site-specifically labeled isotopologues, specifically [1-^13^C,^2^H]glucose, to probe bottlenecks in the upper glycolysis of *L. lactis* (Figure 2B). The accumulating metabolites of upper glycolysis particularly indicate a kinetic barrier at the phosphofructokinase step, as fructose-6-phosphate accumulates significantly, prior to a slower conversion to fructose-1,6-bisphosphate, albeit the latter metabolite had been shown to accumulate on a slower timescale. The slow accumulation of fructose-1,6-bisphosphate can be ascribed to the depletion of NAD^+^ by ongoing glycolytic conversions, which also charge the energy state of the cell in the absence of futile cycles depleting ATP [31,36].

**Figure 3:**
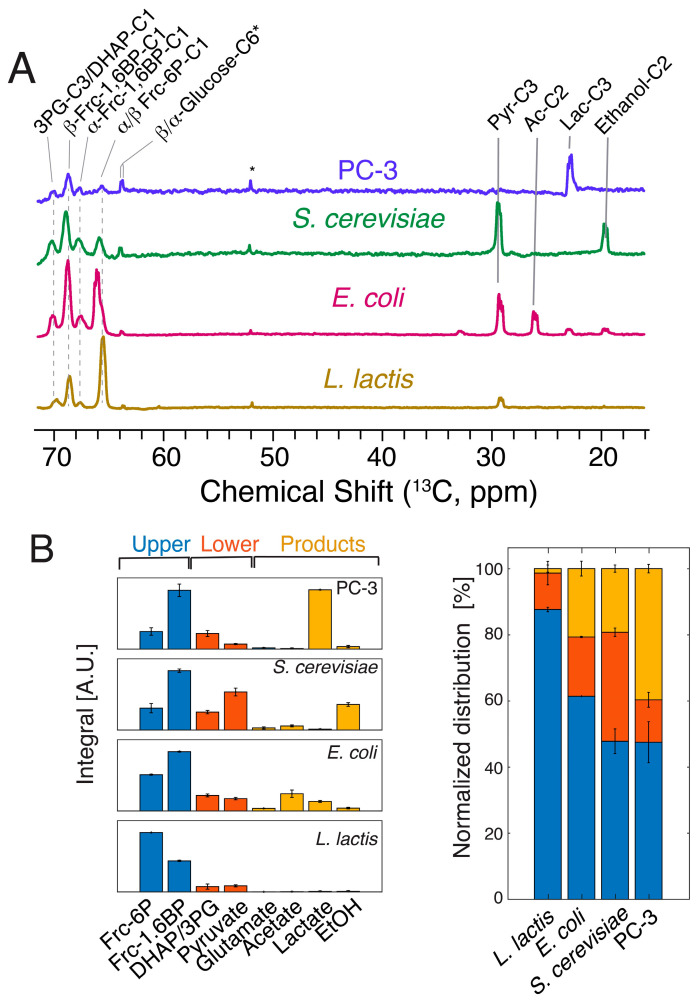
(**A**) 1D ^13^C spectra acquired on various cell cultures three seconds after supplying 6 mM hyperpolarized [1-^13^C,^2^H]glucose to probe the influx of glucose into the metabolic network, with an emphasis on upper glycolysis. A background signal in the preparation is indicated by an asterisk. (**B**) Integration and classification of the spectra displayed in (**A**). Metabolites partition into end products, especially in cancer cells, within 3 s, while increasingly higher fractions of substrate are found in metabolites of upper glycolysis (blue) relative to metabolites of lower glycolysis (red) and end products (orange) for *S. cerevisiae*, *E. coli* and *L. lactis*. Slower glycolysis correlates with lower ratios of fructose-1,6-bisphosphate to fructose-6-phosphate, indicating kinetic control at the phosphofructokinase step especially in *L. lactis*. Experimental variations of signal areas are indicated for biological replicate cell cultures (*n* = 2) of the experiments, as shown in (**B**).

The kinetic barriers in upper glycolysis during metabolic initiation were subsequently compared using hyperpolarized [1-^13^C,^2^H]glucose as a probe molecule in *E. coli*, *S. cerevisiae* and human PC-3 cancer cell lines. These cell types had previously been compared for their thermodynamic profile of glycolysis and absolute metabolite concentrations [1,2]. Both thermodynamics and metabolite concentrations in these cells had indicated similarity between the different cell types, with larger similarity between the eukaryotic cells than between the microbial cells. A comparison of kinetic barriers in upper glycolysis can be drawn from the signals between 60 and 70 ppm in the ^13^C NMR spectrum acquired with one 90° pulse three seconds after a glucose bolus injection (Figure 3A) to obtain maximum signal to noise [8]. The timing of the pulse was based on the observed maximum of the fructose intermediates from the dynamic curve (Figure 2B and Figure S2). These spectra indicated a similar usage of upper glycolysis in *S. cerevisiae* and human PC-3 cancer cell lines, while *E. coli* and especially *L. lactis* show deviating patterns (Figure 3A). 

In summary, hyperpolarized [1-^13^C,^2^H] can be used to afford insight into glucose influx of the central metabolism, including upper glycolysis. Resultant data indicated that the conversion of fructose-6-phosphate to fructose-1,6-phosphate in upper glycolysis is most limited in *L. lactis* and increasingly less limited in *E. coli* and in the eukaryotic cells. Notably, these observations of a metabolic barrier at the phosphofructokinase step were consistent with more indirect findings using genetic methods, which had shown that reduced activity of phosphofructokinase was correlated with reduced growth, glucose consumption, and lactate formation rates in *L. lactis* [37]. 

### 2.3. Influx and Passage through Upper Glycolysis Limits Conversion through the Entire Pathway

The spectra of Figure 3A include signals from metabolic end products in addition to the signals deriving from fructose-phosphates. It was evident that the uptake and conversion of substrate to products within three seconds was largest in eukaryotic cells but decreased in *E. coli* and especially in *L. lactis*. Figure 3B shows that more than 80% of converted glucose remained partitioned to metabolites of upper glycolysis in *L. lactis*, while this fraction was less than 50% in *S. cerevisiae* and PC-3 cells. Correspondingly, the conversion of glucose to end products was negligible in *L. lactis*, while more than 40% of the converted glucose had reacted to product, here lactate, in PC-3 cancer lines within three seconds. Hence, dDNP NMR using site specific isotope labeling indicated that more evolved cells can be optimized to support higher rates of carbohydrate uptake and glycolytic conversion. Notably, the ratio between fructose-1,6-bisphosphate and fructose-6-phosphate, formed rapidly from added glucose, was diagnostic of the overall speed of glucose conversion in glycolysis (Figure 3B and Figure S3) and correlated to the influx of glucose into lower glycolysis end products. These observations indicate that the phosphofructokinase-catalyzed conversion of fructose-6-phosphate to fructose-1,6-bisphosphate proceeds slower in *L. lactis* and *E. coli* than in the tested eukaryotic cells. Likewise, it is noteworthy that fructose-6-phosphate, like 3-phosphoglycerate, triosephosphates and pyruvate, is among the metabolites with highest connectivity in metabolism [33,38,39]. 

In summary, hyperpolarized [1-^13^C,^2^H] not only affords the detection of upper glycolytic intermediates, but also of lower glycolysis and metabolic end products, which allows the correlation of uptake and conversion in upper glycolysis to overall glycolytic progress. In addition, a pattern emerges at wild-type enzyme levels, which indicates that chemicals at highly connected metabolic nodes tend to accumulate for further distribution according to cellular needs. Both the directly observed correlation between overall glycolytic dynamics and the fructose-6-phosphate phosphorylation step [37,40,41], and the observation of varying control of glycolytic dynamics across the tree of life [42] are consistent with independent studies using different methodologies.

### 2.4. Hyperpolarized NMR Using Mutant Strains Shows Altered Kinetics on a Timescale of Seconds, Despite Similar Kinetics on a Timescale of Minutes

After probing the variations in kinetic barriers across cells from different branches of the tree of life, we studied kinetic alterations at individual steps resulting from altered gene expression. Altered gene expression can result in different enzyme activities after synthetic promoters with different strengths are integrated into the genome by site-specific recombination [43]. The strains that were used included strains with enzyme activities of glyceraldehyde 3-phosphate dehydrogenase (GAPDH) or pyruvate kinase (PYK) increased to approximately 200%, in addition to a strain with the activity of triosephosphate isomerase (TPI) reduced to 30%. These strains have previously been reported to support an essentially unaltered glycolytic flux to the lactate end product when compared to the wild type [43]. The strains were hence selected to probe if observations of intracellular glycolysis reveal elusive kinetic alterations along the pathway upon alterations of individual enzyme activities, especially for changes in GAPDH or PYK activity, which are widely considered to be regulatory enzymes in glycolysis. 

**Figure 4:**
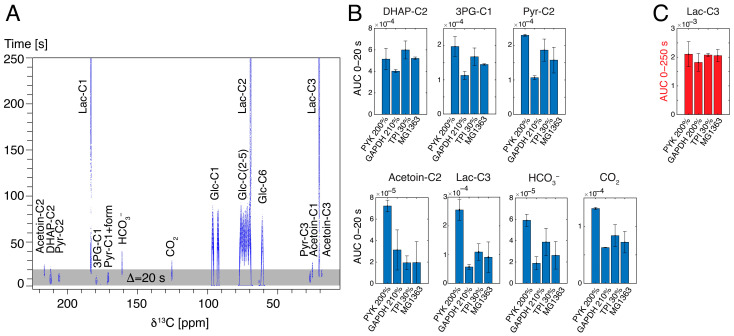
(**A**) Influx of 6 mM hyperpolarized [U-^13^C,^2^H]glucose into the metabolism of wild-type *L. lactis* and window (0–20 s) used to probe pre-steady-state glycolysis in various strains. (**B**) Comparison of influx of 6 mM hyperpolarized [U-^13^C,^2^H]glucose into the metabolism during pre-steady-state glycolysis in various strains of *L. lactis* shows different kinetic behavior on a timescale of 0–20 s, despite approaching similar lactate formation on a longer (steady-state) timescale (**C**). Abbreviations: GAPDH—glyceraldehyde phosphate dehydrogenase; PYK—pyruvate kinase; TPI—triosephosphate isomerase. Experiments were performed for biological replicates and time-resolved data are shown in Figure S4.

Hyperpolarized [U-^13^C,^2^H]glucose was used for real-time assays of the different *L. lactis* strains to evaluate whether different enzyme levels elicit different accumulations of metabolites upstream of kinetic barriers in pre-steady-state conditions. To this end, areas under the curves (AUCs) were recorded in biological replicate cell cultures (*n* = 2) for seven different metabolites. These measurements showed that reduced TPI levels did not affect the accumulation of intermediates and products in the initiation of glycolysis upon a bolus of hyperpolarized [U-^13^C,^2^H]glucose, while increased pyruvate kinase activity indeed facilitated the influx of glucose signals into acetoin and lactate under pre-steady-state conditions (Figure 4). These observations were paralleled by an increased formation of CO_2_ and HCO_3_^–^. A more complex pattern emerged for the strain overexpressing GAPDH, which indicated a surprising decline in the influx of metabolites from upper glycolysis into lower glycolysis, possibly due to dysregulation in cofactor pools because of altered enzyme levels. By contrast, the formation of lactate within five minutes of feeding with a 6 mM glucose pulse validated the identical formation of the product in homolactic fermentation of all four *L. lactis* strains (Figure 4C and Figure S4). Although all strains reached similar final levels of lactate, as previously described in the literature, the initial influx of lactate and metabolic intermediates occurred at different rates. Hence, dDNP NMR of central metabolism in *L. lactis* provides kinetic insight into a phenotype that is not accessible by more routine methods. These analytical capabilities may hence lay the foundation for future mechanistic studies which can provide an experimental understanding of metabolic fluxes in dependence on enzyme activities and cofactor pools.

### 2.5. In-Cell Spectroscopic Visualization of Response to Enzymatic Inhibitor and to Altered Carbon Source

**Figure 5:**
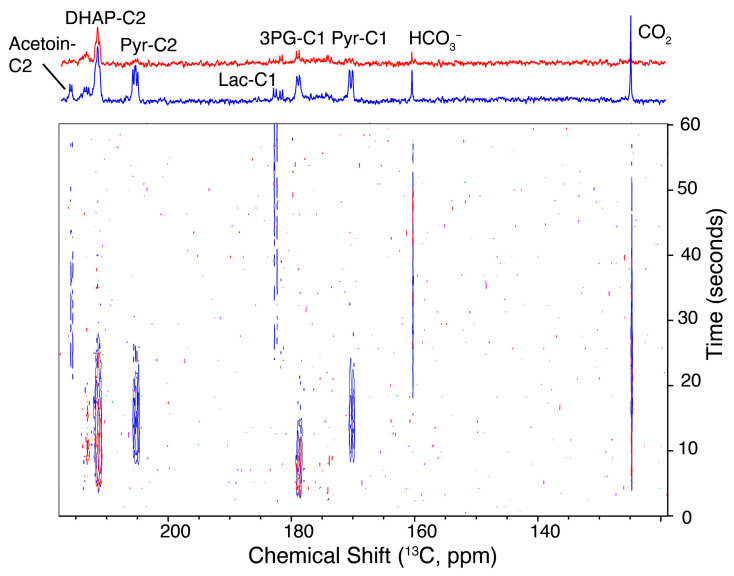
Influx of 6 mM hyperpolarized [U-^13^C,^2^H]glucose into the metabolism of wild-type *L. lactis* to visualize the metabolic effects of exposing *L. lactis* to 200 μM iodoacetate, an inhibitor of GAPDH, for 15 min (red spectra). Such exposure obstructs influx into metabolites of lower glycolysis and products, while still allowing some influx into metabolites upstream of GAPDH. Time series (**bottom**) and sum spectra during 60 seconds of glucose influx (**top**) are compared in the absence (blue) and in the presence (red) of 200 μM iodoacetate.

Following the assays showing the effects of different genomes and of different enzyme activities on kinetic barriers in glycolysis, we evaluated if medium composition, nutrition and inhibitors can elicit significant effects on the accumulation of kinetic intermediates in the glycolysis of *L. lactis*. These measurements were motivated by the fact that *L. lactis* experiences significantly variable media composition during biotechnological processes. Minor changes in nutrition have been implicated in big metabolic effects [44], as for instance in substrate mixtures [4,22,45], and increases in nutrients can elicit significant changes on a par with genetic engineering, yielding drastic changes in pathway usage [4]. Initially, we attempted to visualize the effects of inhibiting individual enzyme activity in a time-resolved manner using 6 mM hyperpolarized [U-^13^C,^2^H]glucose substrate. Sufficiently inhibited enzyme activities are expected to obstruct glycolytic kinetics, an effect that also had been observed for low-level expressions of glycolytic enzymes [43]. We explored the effect of exposing *L. lactis* to 200 μM iodoacetate for 15 min, since iodoacetate is known for its ability to irreversibly inhibit GAPDH [46], prior to observing intracellular kinetics. The resultant time-resolved spectra for pre-steady-state glycolysis are shown in Figure 5 and the reproducibility is indicated in Figure S5. Experiments in the presence of iodoacetate show that glucose indeed can partition to metabolites upstream of GAPDH, specifically DHAP. By contrast, the absence of conversion to the metabolites of lower glycolysis (such as pyruvate) and of excreted products (such as acetoin) is evident in the presence of iodoacetate. These observations somewhat reflect observations of treatment responses in human cells with hyperpolarized NMR [47,48], and indicate that functional screening of xenochemicals can pinpoint sites of action in microorganisms.

**Figure 6:**
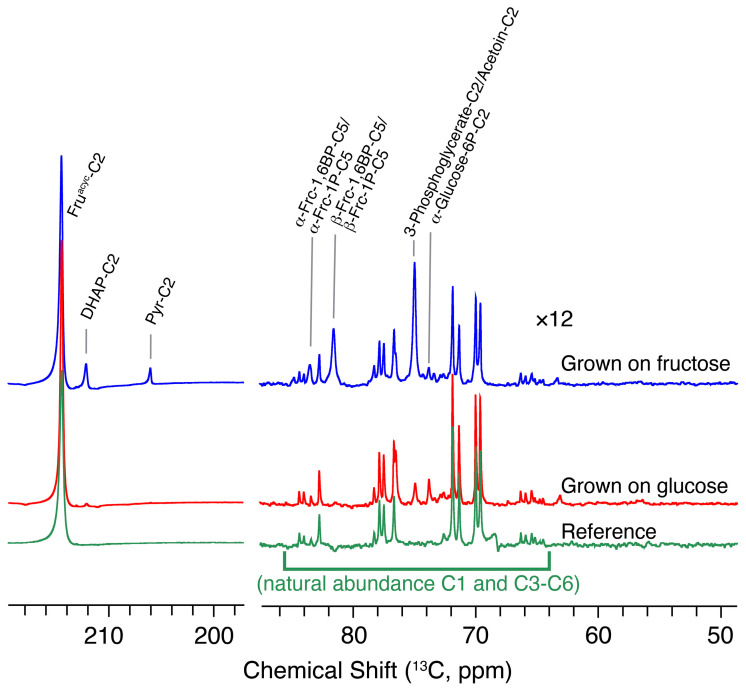
Metabolic adaptation of *L. lactis* to growth on either glucose (red) or fructose (blue) as the carbon source. Influx of hyperpolarized [2-^13^C]fructose into cellular metabolites is shown for both cases using a 90° excitation pulse after three seconds of injection to cell suspension, alongside a reference spectrum of the hyperpolarized [2-^13^C] fructose (green) without *L. lactis* cells (yielding a weak background of protonated C1 and C3–C6 positions from fructofuranoses and fructopyranoses). Signals below 90 ppm are scaled by a factor of 12 relative to signals above 195 ppm.

We then evaluated nutritional effects by growing *L. lactis* on different carbon sources, specifically on fructose in addition to glucose. *L. lactis* has an inducible fructose phosphotransferase system that produces fructose 1-phosphate entering EMP glycolysis as fructose 1,6-bisphosphate [49]. In these experiments, a 90° excitation pulse was again used to detect the influx of site-specifically labeled substrate into EMP glycolysis for three seconds (Figure 6, experimental reproducibility shown in Figure S6). The inducible nature of the phosphotransferase system was validated by the increased influx of fructose into the EMP glycolysis for cells grown on fructose rather than on glucose (Figure 6; 7.9-fold increased influx into DHAP). These experiments validated that metabolites of upper glycolysis can be probed using site-specifically labeled carbohydrates. In addition, the experiments provided a positive identification of 3-phosphoglycerate as a main intermediate. Such an identification is useful, as the signal near 181.3 ppm when using [U-^13^C,^2^H]glucose could derive from either 3-phosphoglycerate in EMP glycolysis or from 6-phosphogluconate in the oxidative pentose phosphate pathway. The positive identification of 3-phosphoglycerate was consistent with the absence of a signal near 181.3 ppm in the conversion of [1-^13^C,^2^H]glucose, which indicates that this signal is not 6-phosphogluconate formed in the pentose phosphate pathway.

### 2.6. Glucose/Pyruvate Substrate Mixtures Direct Glucose Carbon towards Acetoin

We finally expanded on the effect of the carbon source to explore the effect of substrate mixtures on the metabolism of *L. lactis* wild type. Specifically, we wanted to further explore the ability of *L. lactis* to direct glucose in resting cells predominantly into acetoin. We hypothesized that this influx, which we had previously detected using hyperpolarized NMR experiments, results from the accumulation of pyruvate upon the sudden availability of glucose and from the limited availability of NADH in resting cells prior to reaching steady-state conditions. Similar behavior has been described for *S. cerevisiae*, where limited availability of NADH likewise can enforce the overflow of pyruvate into acetoin under suitable conditions [4]. The formation of lactate from pyruvate stoichiometrically converts NADH to NAD^+^ and is more purposeful than acetoin formation under reducing conditions. By contrast, overflow into acetoin should be favored when supplementing glucose substrate with the more oxidized pyruvate substrate, which itself can be biotechnologically produced via aerobic carbohydrate metabolism [50] or from lactate [51].

**Figure 7:**
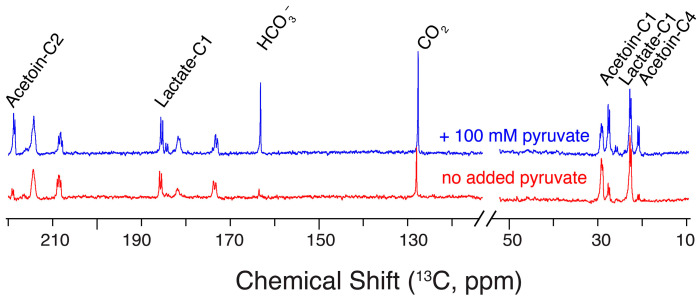
Comparison of the effect of substrate mixture on *L. lactis* metabolism as measured using dDNP NMR to probe the influx of 6 mM hyperpolarized [U-^13^C,^2^H]glucose into EMP glycolysis within 50 s. Added pyruvate in a glucose/pyruvate substrate mixture enhanced glucose influx into acetoin.

**Figure 8:**
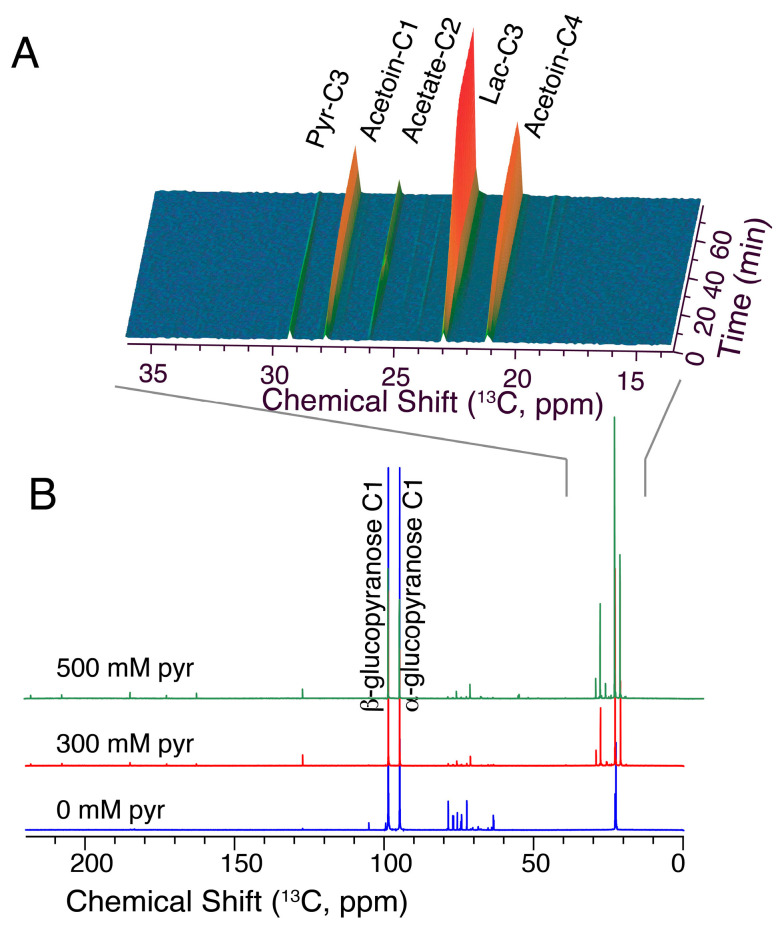
Compositional changes to product mixtures in the presence of glucose/pyruvate substrate mixture. Conventional real time ^13^C NMR was used to follow the conversion of 100 mM [1-^13^C]glucose in the presence of variable concentrations of pyruvate on the minutes timescale ((**A**): time course from experiment with 500 mM added pyruvate; (**B**): sum of spectra over 60 min experiment time in the presence of 0, 300 and 500 mM pyruvate).

Repeating hyperpolarized NMR assays in the presence of 100 mM exogeneous pyruvate indicated a more efficient and faster influx of glucose-derived carbon into acetoin: the influx of glucose into acetoin, CO_2_ and HCO_3_^–^ was increased in the presence of 100 mM exogeneous pyruvate during the initiation of glycolysis by a bolus of hyperpolarized [U-^13^C,^2^H]glucose (Figure 7). Thus, the ratio of the acetoin C2 signal relative to the lactate C1 signal increased from 0.27 ± 0.08 in the absence of added pyruvate to 1.17 ± 0.08 in the presence of 100 mM exogeneous pyruvate (with errors deriving from biological replicates with *n* = 2). Previous studies using 21 mM pyruvate/9 mM glucose substrate mixtures had indicated changes to glycolysis and fermentation in resting citrate-positive *L. lactis* using conventional NMR detection, albeit negligible formation of acetoin from glucose had been described under these conditions [52]. Beyond providing insight into glycolytic dynamics and responses, the modulation of EMP glycolysis to provide C4 products such as acetoin from non-fossil resources has attracted considerable attention [50,53,54,55] and has included approaches for biotechnological production in *L. lactis* strains lacking lactate dehydrogenase [54]. Hence, we were interested in tracking acetoin product accumulation with wild-type *L. lactis* under steady-state conditions on a minutes-to-hours timescale using glucose/pyruvate substrate mixtures at intensified conditions with high carbohydrate and pyruvate concentrations. Resultant product mixtures were characterized using 100 mM [1-^13^C]glucose in conventional NMR assays (Figure 8) in the absence and in the presence of external pyruvate. External pyruvate was added to final concentrations of 300 mM and 500 mM in addition to a reference experiment in the absence of added pyruvate. 

**Figure 9:**
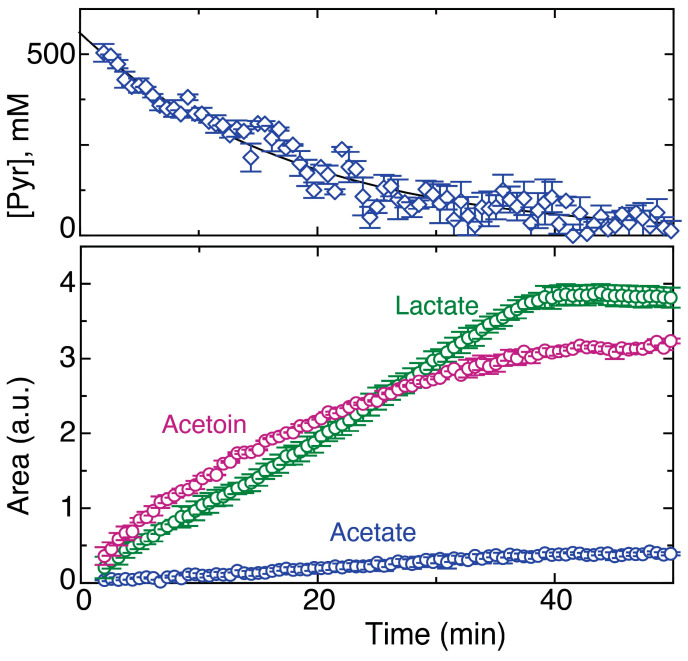
Time course of the influx of the C1 carbon of 100 mM [1-^13^C]glucose in the presence of 500 mM unlabeled pyruvate into acetoin (sum of C1 and C4 signals), lactate and acetate. The corresponding time course of pyruvate decay as tracked by signal areas at natural ^13^C isotope abundance is shown in the top panel. Experimental errors for biological replicate cell cultures (*n* = 2) are shown.

The presence of oxidized pyruvate co-substrate indeed shifts the purely homolactic metabolism (Figure 8B, blue spectrum) towards the formation of acetoin and acetate (Figure 8A,B, red and green spectra). In addition, the conversion of glucose is stimulated by the presence of pyruvate (see for instance the natural abundance of C6 glucose signals near 62 ppm in Figure 8B), thus supporting the notion that glycolytic progress can be limited by the availability of NAD^+^ [31,56]. These product signals deriving from [1-^13^C]glucose were integrated and compared to the decay of the external pyruvate. The integrations showed that acetoin becomes a predominant product at sufficiently high concentrations of pyruvate (>300 mM), where the initial rate reflects 24 ± 4% faster influx of C1 carbon of 100 mM [1-^13^C]glucose into acetoin than into lactate. Selectivity of acetoin formation decreases along the reaction pathway (Figure 9) with decreasing pyruvate concentrations. As acetoin usually is a minor fermentation product [57], the formation of acetoin from a glucose/pyruvate mixture can thus be increased ~400-fold relative to its formation in the absence of added pyruvate without genetic engineering. Notably, the glucose/pyruvate substrate mixture was well tolerated even in growing MG1363 cells. A growth experiment showed that the addition of 100 mM pyruvate increased growth by 77% and the addition of 300 mM increased growth by 23%, while the addition of 500 mM pyruvate reduced growth by 85% compared to the absence of pyruvate addition. Overall, these observations indicated that suitable substrate mixtures can be designed based on insight into the response of *L. lactis* to limited NADH availability in pre-steady-state glycolysis. Use of lactate-derived pyruvate in addition to glucose substrate appears to be a promising means of complementing the genetic engineering of *L. lactis* for the formation of bio-sourced chemicals beyond lactate.

## 3. Materials and Methods

### 3.1. Chemicals and Cells

Chemicals were purchased from Merck Sigma-Aldrich (St. Louis, MO, USA) unless stated otherwise. [1-^13^C,^2^H]glucose was purchased from Cambridge Isotope Laboratories (Tewksbury, MA, USA). *Escherichia coli* MG1655 (ATCC 700926) and the prostate cancer cell line PC-3 (ATCC CRL-1435) were purchased from American Type Culture Collection (ATCC, Manassas, VA, USA). Dry yeast from Seitenbacher (Buchen, Germany) was purchased via online retail. *Lactoccocus lactis* MG1363 and three strains with altered enzyme activities in TPI, GAPDH and PYK were provided by the collection of one of the authors (Peter Ruhdal Jensen and group, Technical University of Denmark, Kgs. Lyngby, Denmark).

### 3.2. Cell Culture

*L. lactis* was grown in M17 medium supplemented with 1% glucose. An overnight culture was diluted to a turbidity at 600 nm wavelength OD_600_ = 0.05 in 90 mL M17 medium in a 100 mL blue cap flask and growing biological replicate cultures (*n* = 2) at 303 K with shaking at 120 rpm until OD_600_ = 0.5. Then, 30 ml cell suspension was harvested by centrifugation (5000× *g* for 5 min), the cell pellet was washed with 5 ml MES buffer (30 mM, pH 5.65) and was centrifuged again at 5000× *g* for 5 min. The cell pellet was hereafter suspended to a total volume of 250 μL with MES buffer and 200 μL of the cell suspension was transferred to a Shigemi NMR tube and fitted with an injection line before insertion into the NMR magnet.

For growth experiments in the presence of pyruvate, *L. lactis* was grown in M17 medium supplemented with 1% glucose. Two batches of growth medium were prepared: (A) without addition of pyruvate and (B) with 1 M sodium pyruvate. 1.8 mL growth medium was prepared with final concentrations of 0, 100, 300 and 500 mM pyruvate from stock solutions A and B in 2 mL Eppendorf tubes. To each tube, 18 μL of an overnight culture was added, and the cells were grown at 303 K with shaking at 120 rpm. After 18 h, the cell density (OD_600_) was measured, showing an approximately tenfold decline in growth in the presence of 500 mM pyruvate (OD_600_ = 0.2) in comparison to the presence of 100 mM pyruvate (OD_600_ = 2.3).

*E. coli* was grown in tryptone soy broth (TSB). An overnight culture was diluted to OD_600_ = 0.02 in 50 mL TSB medium in a 250 mL conical flask and grown at 310 K with shaking at 200 rpm under an aerobic environment until OD_600_ = 1.0. At OD_600_ = 1.0, 30 mL cell suspension was harvested by centrifugation (5000× *g* for 5 min), the cell pellet was washed with 5 mL HEPES buffer (40 mM, pH 7.0) and centrifuged again (5000× *g* for 5 min). The cell pellet was hereafter suspended in 300 μL HEPES buffer and 200 μL of the cell suspension was transferred to a Shigemi NMR tube and fitted with an injection line before insertion into the NMR magnet.

PC-3 cells were cultivated in RPMI 1640 medium (Gibco, Carlsbad, CA, USA) containing 4.5 g/L glucose, sodium pyruvate, L-glutamine, HEPES, 1% penicillin and streptomycin solution, and 10% fetal bovine serum (FBS) at 310 K in 5% CO_2_ atmosphere. The cells were passaged every 3–4 days after harvest with trypsin. In a T175 flask 1.0 × 10^6^ cells were seeded and after 6 days the confluent cell layer was harvested by trypsinization and subsequently resuspended in 10 mL PBS (with Ca^2+^ and Mg^2+^) and counted with a cell counter. The volume corresponding to 1.5 × 10^7^ cells was centrifuged (200 rpm, 3 min) and the cell pellet was resuspended to a total volume of 200 μL with HEPES buffer. The cell suspension was transferred to a Shigemi NMR tube and fitted with an injection line before insertion into the NMR magnet.

*S. cerevisiae* suspensions were prepared as previously described [5]. In brief, 100 mg dry yeast was suspended in 550 μL MES buffer. From this suspension, 200 μL was transferred to a Shigemi NMR tube and fitted with an injection line before insertion into the NMR magnet.

### 3.3. Inhibition of Glycolysis with Iodoacetate

For inhibition of glycolysis with iodoacetate (IA), *L. lactis* was prepared as described above. Immediately before transfer to the NMR tube, 10.5 μL of an IA stock (4.8 mM in Milli-Q water) was added to the cell suspension. From this suspension, 200 μL was transferred to a Shigemi NMR tube and fitted with an injection line before insertion into the NMR magnet. It took 8 min from addition of IA to injection of hyperpolarized glucose. The IA concentration was 200 μM during the 8 min incubation and 90 μM during NMR detection.

### 3.4. Fructose as Carbon Source and dDNP Substrate

*L. lactis* was either grown with glucose as the main carbon source as described above or with fructose as the main carbon source. For growth on fructose as the main carbon source, 1% fructose was added to the M17 medium instead of glucose. *L. lactis* was hereafter grown as described for the experiments using glucose substrate.

### 3.5. Pyruvate/Glucose Substrate Mixtures 

Cells were prepared as described above. In the last resuspension step after washing, part of the buffer was replaced with a pyruvate stock solution made in the corresponding buffer. A 1.2 M sodium pyruvate solution was made in the respective buffer (MES or HEPES). To obtain 100 mM pyruvate in the NMR tube, the cell pellet was resuspended in 203 μL buffer with 47 μL pyruvate stock. From this suspension, 200 μL was transferred to a Shigemi NMR tube and fitted with an injection line before insertion into the NMR magnet. It took 8 min from addition of pyruvate to injection of hyperpolarized glucose.

### 3.6. NMR Experiments

Conventional NMR experiments were acquired at 303 K on a Bruker (Fällanden, Switzerland) 800 MHz NMR instrument equipped with a TCI Cryoprobe and a SampleJet sample changer. A time series of one-dimensional ^13^C spectra was implemented by accumulating 16 transients per time point. Each FID was sampled by acquiring 32768 complex data points during an acquisition time of 682 milliseconds. Up to 128 data points were acquired with a time resolution of 0.59 min per time point. The time series was implemented as a pseudo-2D NMR experiment acquiring a 2D NMR data set consisting of a series of 1D NMR spectra in real time. Shimming, tuning and matching as well as pulse calibration were conducted on a dummy sample in the absence of isotope-labeled glucose. Experiments commenced by mixing 10 mg [1-^13^C]glucose in 50 μL D_2_O and a cell suspension of 550 μL volume in 30 mM MES buffer (pH 5.6). The time between mixing of substrate with cell suspension and the start of NMR data acquisition was approximately 1.5 min. Experiments comparing the effects of glucose/pyruvate substrate mixtures were conducted in the presence of up to 500 mM pyruvate (final concentration) in 3 mm NMR tubes. Signals deriving from [1-^13^C]glucose were integrated, namely C1 and C4 positions in acetoin, C2 in acetate and C3 in lactate. That these positions predominantly (>94%) reflect [1-^13^C]glucose metabolism rather than metabolism of natural abundance pyruvate was validated by comparison to the alcohol positions in acetoin and lactate, and by comparison to a reference experiment acquired in the absence of [1-^13^C]glucose (Figure S7).

### 3.7. DNP 

[1-^13^C,^2^H]glucose, [U-^13^C,^2^H]glucose or [2-^13^C]fructose were prepared for DNP as follows: 92 mg isotope-labeled glucose was mixed with 70 mg MilliQ water, 4.1 mg OX063 (GE Healthcare, Waukesha, WI, USA) and 2.6 mg gadoteridol (stock 50 μmol/mL in MilliQ water, Bracco Imaging, Milan, Italy). 30 mg of this sample preparation was loaded to a sample cup and transferred to a Hypersense polarizer operating at 3.35 T (Oxford Instruments, UK). After approximately 60 min of polarization, a solid-state polarization of 30% was obtained and the sample was dissolved in 5 mL buffer (MES or HEPES). From this dissolved sample, 250 μL was injected into the cell suspension placed in the NMR magnet, yielding 6 mM hyperpolarized glucose concentration. The procedure is detailed in the Supplementary Materials and in excellent reviews, such as [58,59].

### 3.8. dDNP NMR

Cell suspensions in an NMR tube were placed into a 500 MHz Bruker spectrometer equipped with a 5 mm DCH CryoProbe and thermally equilibrated to 303 K or 310 K. Metabolism was followed using one of two different acquisition strategies: (i) A time series of ^13^C NMR spectra was implemented as a pseudo-2D experiment using a 10° excitation pulse. Each ^13^C spectrum in the time series was acquired with 11264 complex data points (sampling the FID for 344 ms) every 0.5 s; (ii) One-pulse experiments with maximum sensitivity were acquired using a 90° excitation pulse. The ^13^C spectrum was acquired with 11,264 complex data points (sampling the FID for 344 ms). The timing of the pulse was determined as the maximum integral obtained with a time series of 10° excitation pulses as described for acquisition strategy (i). We note that full reproducibility of the polarization level between dDNP experiments may not be warranted. We have previously determined a standard deviation of signal areas for technical replicates below 8% by repetition in quadruplicate on suspensions of commercial dry yeast, using the same experimental setup for in-cell dDNP NMR assays as used herein [8].

### 3.9. Data Analysis and Fitting

All spectra were processed using Bruker TopSpin 4.1.4 and were analyzed using the same software. Spectra were zero-filled to twice the number of acquired points. Integration of pseudo-2D data was performed using the Dynamics Applications module of TopSpin. Data were plotted using pro Fit 7 (Quansoft, Zurich, Switzerland) or Matlab R2023a (Mathworks, MA, USA).

## 4. Conclusions

In conclusion, we find that the dynamics of glucose metabolism on a timescale of seconds can be observed in *L. lactis* under in vivo conditions using hyperpolarized NMR. The approach provides information that is complementary to other methods, which characterize metabolic dynamics and regulation by slower measurements, modeling, and ex cellulo assays. Thus, dDNP NMR of central metabolism in *L. lactis* provides molecular phenotypes that can aid future mechanistic studies of metabolic fluxes dependent on enzyme activities, gene knockouts or cofactor pools. The sudden availability of glucose in the *L. lactis* wild-type strain MG1363 elicits overflow towards an acetoin-forming pathway that is used at low NADH/NAD^+^ ratios. This pathway to bio-sourced acetoin can be further enforced by the presence of exogeneous pyruvate as a substrate that is more oxidized than glucose and can elicit a conversion of glucose into acetoin that exceeds the conversion to lactate in the wild-type strain at steady state. The initiation of glycolysis in the presence of suddenly available glucose in *L. lactis* is slower than in *E. coli* but is faster in eukaryotic *S. cerevisiae* and human prostate cancer cells (PC-3). Kinetic barriers in the upper parts of glycolysis were visualized and compared in these different cells, showing that slower conversion in glycolysis was correlated to a slower influx of glucose into fructose-1,6-phosphate pools in *L. lactis*, consistent with a previously suggested correlation between the phosphofructokinase-catalyzed step and overall glycolytic dynamics [41,42]. Kinetic changes to enzyme-catalyzed steps were likewise evident in *L. lactis* strains with increased or reduced activity of individual glycolytic enzymes. Despite a comparable steady-state formation of lactate, a faster influx into both acetoin- and lactate-forming pathways was thus apparent in strains with doubled activity of pyruvate kinase. Such insights into dynamic processes in complex systems hinge on sufficiently rapid time-resolved observations rather than endpoint measurements. Overall, the use of hyperpolarized NMR probing of glycolytic dynamics in Gram-positive production strains such as *L. lactis* provides unique insight into metabolic kinetics and their regulation inside the living cell.

## Data Availability

The original contributions presented in the study are included in the article/supplementary material. Further inquiries can be directed to the corresponding authors.

## Supplementary Materials

The following supporting information can be downloaded at https://www.mdpi.com/article/10.3390/molecules29174133/s1: Figure S1: Outline of dDNP NMR instrumentation; Figure S2: Dynamic profile of fructose-6-phosphate obtained with a series of 10° pulses; Figure S3: Ratio of Frc-1,6BP and Frc-6P formed within 3 s in *L. lactis*, *E. coli*, *S. cerevisiae* and PC-3 cells; Figure S4: Dynamic profile of lactate and other metabolites obtained with a series of 10° pulses; Figure S5: Reproducibility when probing influx of hyperpolarized [U-^13^C,^2^H]glucose in the presence of 200 μM iodoacetate; Figure S6: Reproducibility of observing the influx of hyperpolarized [2-^13^C]fructose into cellular metabolites; Figure S7: Reference experiment showing that observed metabolites using [1-^13^C]glucose substrate and conventional NMR derive from the glucose rather than from natural abundance pyruvate.
