# Supplementary material for: Hyperpolarized 13C NMR Reveals Pathway Regulation in Lactococcus lactis and Metabolic Similarities and Differences Across the Tree of Life
Source: Molecules. 2024 Aug 30;29(17):4133. doi: 10.3390/molecules29174133 (PMC11397382; doi:10.3390/molecules29174133)
Supplement: Supplementary file 1 [file molecules-29-04133-s001.zip › molecules-3122611-supplementary.pdf]

Supplementary Information

Hyperpolarized ^{13}C NMR Reveals Pathway Regulation in *Lactococcus Lactis* and Metabolic Similarities and Differences Across the Tree of Life

Sebastian Meier[1*], Alexandra L.N. Zahid[2], Lucas Rebien Jørgensen[2], Ke-Chuan Wang[2], Peter Ruhdal Jensen[3], and Pernille Rose Jensen[2*]

[1]Department of Chemistry, Technical University of Denmark, 2800 Kgs. Lyngby, Denmark
[2]Department of Health Technology, Technical University of Denmark, 2800 Kgs. Lyngby, Denmark
[3]Department of National Food Institute, Technical University of Denmark, 2800 Kgs. Lyngby, Denmark
*Correspondence: semei@kemi.dtu.dk, peroje@dtu.dk

Detailed description of dDNP-NMR experiment

In the dDNP-NMR experiment, a signal enhancement of more than four orders of magnitude is obtained on a chosen substrate compared to conventional NMR. This vast signal enhancement allows fast reaction kinetics to be followed with subsecond time resolution. The signal enhancement results from the formation of a non-equilibrium state that returns to equilibrium with the longitudinal relaxation time T_1. The longitudinal relaxation time T_1 is on the order of 15-60 seconds for ^{13}C nuclei, depending on the substrate and its isotopic composition. In a dDNP-NMR experiment, the substrate is first polarized in a dedicated instrument called a polarizer (Figure S1). This polarization process is accomplished by mixing the substrate with a stable, non-toxic radical and driving the transfer of spin polarization from electrons to nuclei at 1.3 K temperature with microwaves. After completion of the polarization process, a steady-state is achieved. Molecules with enhanced polarization are now quickly transferred from the solid state at 1.3 K to liquid state at 323 K by flushing the hyperpolarized sample out of the polarizer with 5 ml hot dissolution buffer (starting temperature of the buffer is 453 K). The result is 4.5 ml liquid solution containing the hyperpolarized substrate as a solute. This harvested liquid solution of hyperpolarized substrate is drawn into a syringe and injected into the cell suspension via an injection line. Due to the cold temperature in the polarizer and due to passage through a long and thin injection line, the temperature of the substrate is 303-310 K when it is mixed with the cells. Further experimental details and considerations about the dDNP NMR experiment can be found in literature, such as [1,2].

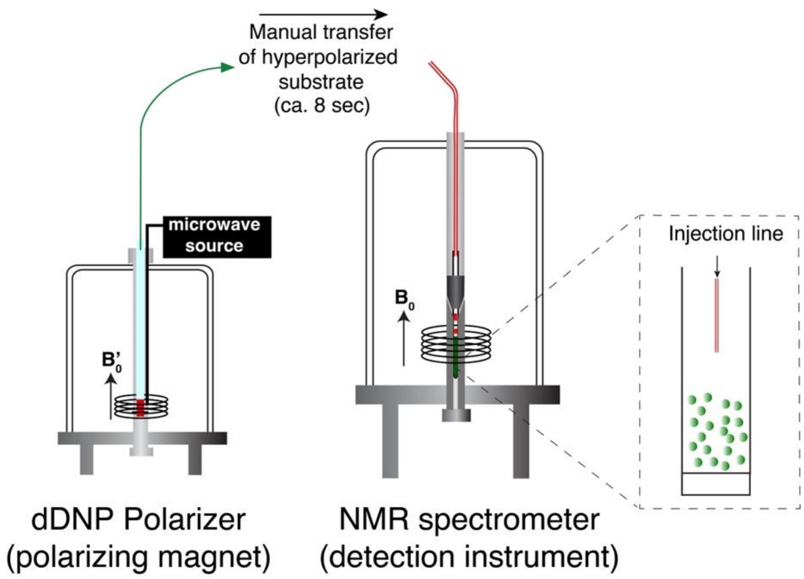

Figure S1. Outline of dDNP NMR instrumentation. The signal enhancement is obtained in the polarizer at 1.3 K. The sample is washed out with hot buffer and the substrate solution is quickly transferred to a cell suspension waiting in the NMR spectrometer fitted with an injection line.

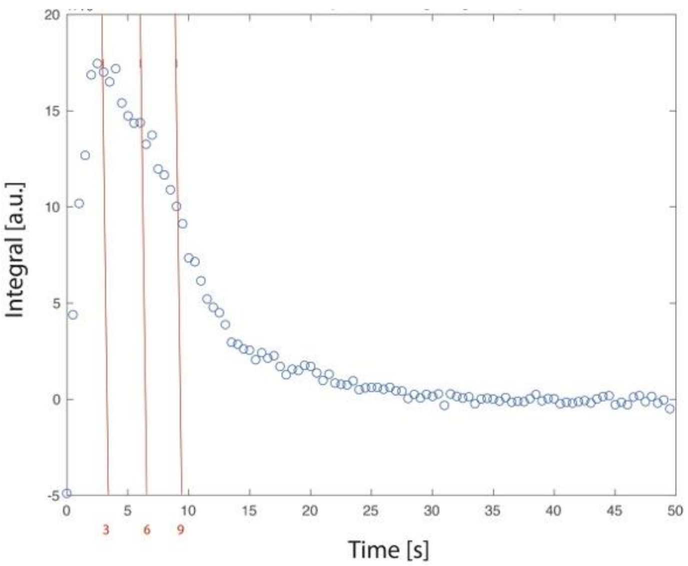

Figure S2. Timing of 90° pulse for detection of intermediates from upper glycolysis. The dynamic profile is shown of fructose-6-phosphate obtained with a series of 10° pulses every 0.5 s after injection of 12 mM hyperpolarized [1-^{2}H,^{13}C]glucose into a cell suspension of wild type *L. lactis* MG1363.

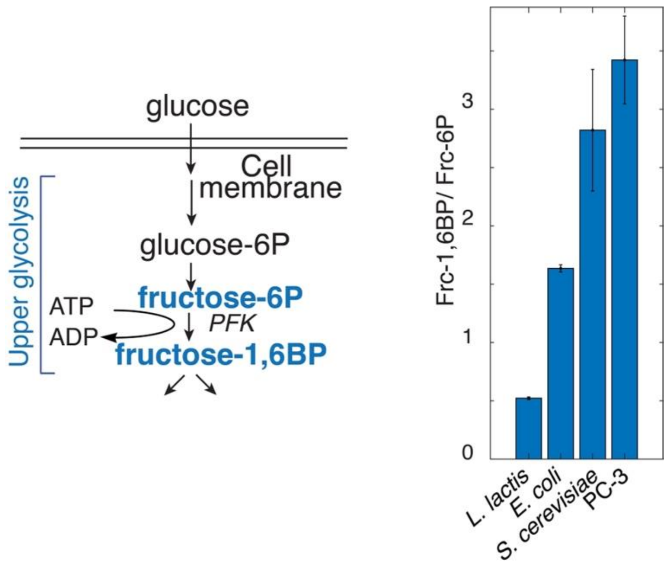

Figure S3. Ratio of Frc-1,6BP and Frc-6P formed within 3 seconds in *L. lactis*, *E. coli*, *S. cerevisiae* and PC-3 cells. This ratio correlated with faster influx into metabolites of lower glycolysis and into fermentation products (biological replicates, n = 2).

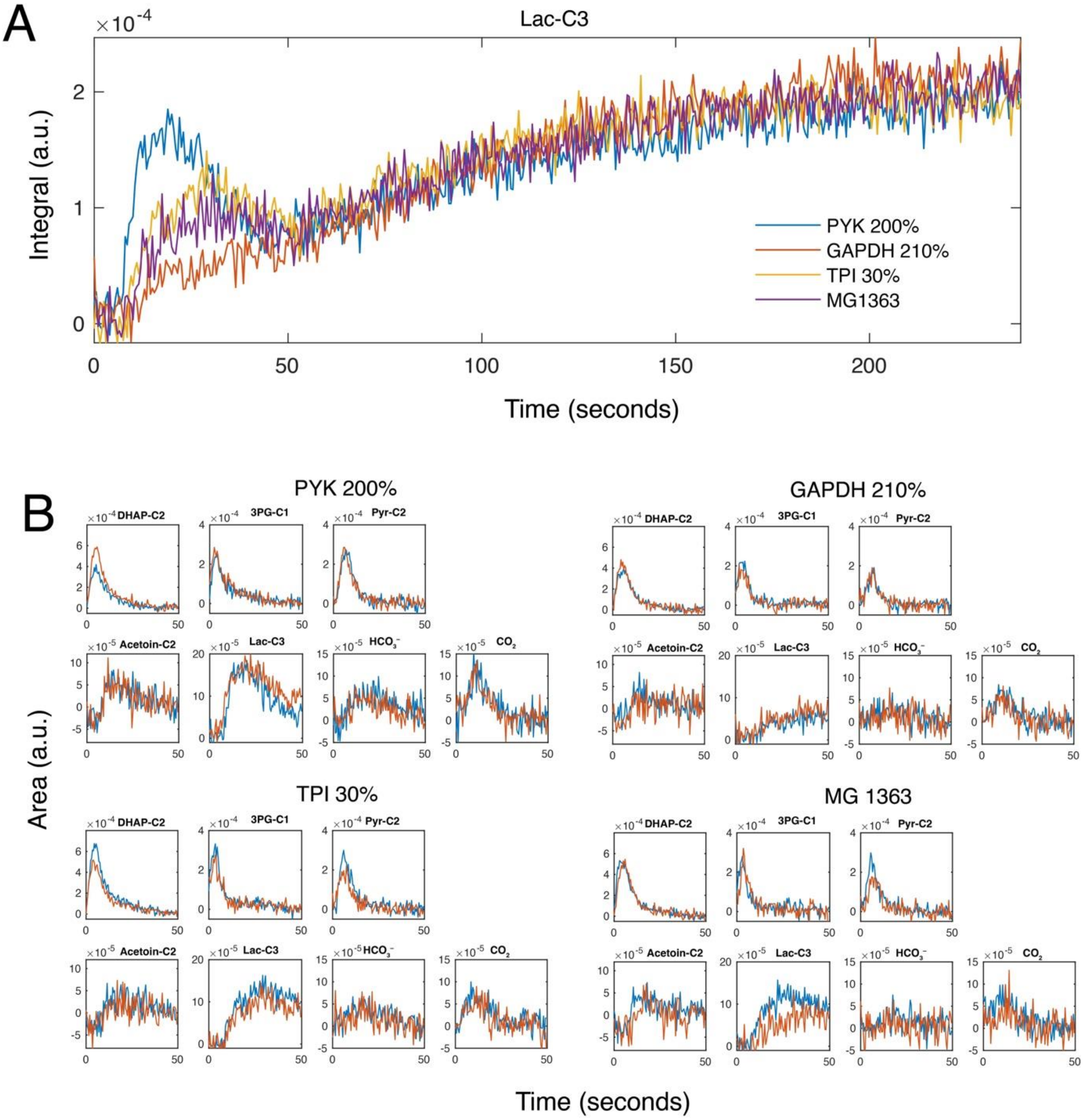

Figure S4. (A) Dynamic profile of lactate-C3 obtained with a series of 10° pulses every 0.5 s after injection of 6 mM hyperpolarized [U-^{2}H,^{13}C]glucose into four strains of *L. lactis*. The curves are an average of two biological replicates for each strain. Note differences on the seconds time scale and similarities on the minutes time scale. Although all strains reach similar final levels of lactate, as previously described in the literature, initial influx into lactate occurs at different rates and is fastest (and hence observed before hyperpolarization fades between 0-50 seconds), in the strain with overexpressed PYK activity. Hence, hyperpolarized NMR of central metabolism in *L. lactis* provides kinetic insight in a phenotype that is not accessible by more routine methods. (B) Dynamic profile of various metabolites in range of 0-50 seconds. The areas under the curves of these data (0-20 seconds) are shown in main text Figure 4. Time profiles for the two biological replicate cultures per strain are shown.

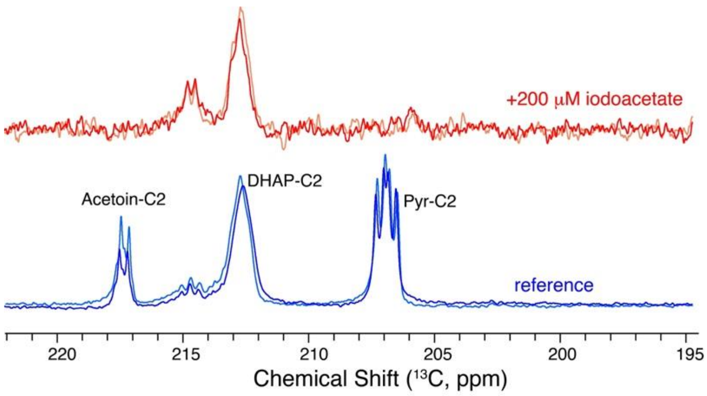

Figure S5. Reproducibility (n = 2) when probing influx of 6 mM hyperpolarized [U-^{13}C,^{2}H]glucose into the metabolism of wild type *L. lactis* in biological replicates to visualize metabolic effects of exposing *L. lactis* to 200 μM iodoacetate (red spectra). The exposure obstructs influx into metabolites of lower glycolysis and products, while still allowing some influx into metabolites upstream of GAPDH. Reproducibility in sum spectra during 60 seconds of glucose influx are compared in the absence (blue) and in the presence (red) of 200 μM iodoacetate.

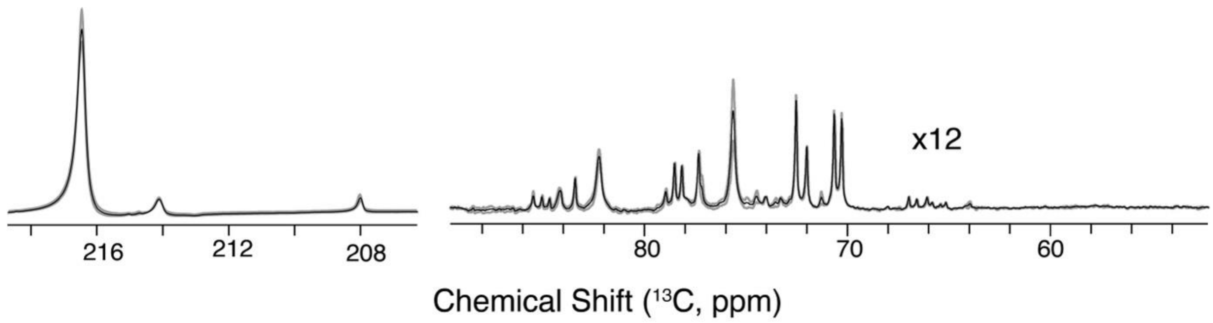

Figure S6. Reproducibility of observing the influx of hyperpolarized [2-^{13}C]fructose into cellular metabolites is shown for both cases using a 90° excitation pulse after three seconds of injection to cell suspension (main text Figure 6; biological replicate cultures with $n = 2$).

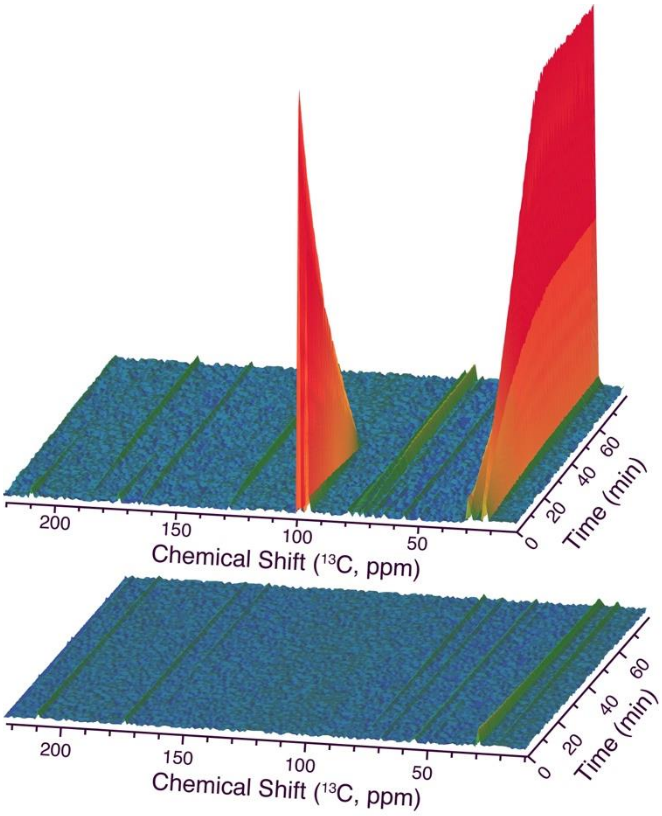

Figure S7. (Top) Time series of ^{13}C NMR spectra the conversion of 100 mM [1-^{13}C]glucose in the presence of 500 mM pyruvate to lactate, acetoin and acetate (in 30 mM MES buffer containing, pH 5.6) alongside (bottom) a reference experiment in the absence of 100 mM [1-^{13}C]glucose that validates that the observed metabolites reflect glucose conversion rather than formation from natural abundance pyruvate.

References

1. Lee, J.H.; Okuno, Y.; Cavagnero, S. Sensitivity Enhancement in Solution NMR: Emerging Ideas and New Frontiers. *Journal of Magnetic Resonance* **2014**, *241*, 18–31, doi:10.1016/j.jmr.2014.01.005.
2. Elliott, S.J.; Stern, Q.; Ceillier, M.; El Daraï, T.; Cousin, S.F.; Cala, O.; Jannin, S. Practical Dissolution Dynamic Nuclear Polarization. *Progress in Nuclear Magnetic Resonance Spectroscopy* **2021**, *126–127*, 59–100, doi:10.1016/j.pnmrs.2021.04.002.
